# Diagnostic and prognostic utility of salivary and serum procalcitonin, interleukin-6, and interleukin-10 in pediatric pneumonia: a prospective case-control study

**DOI:** 10.3389/fped.2025.1627451

**Published:** 2025-09-11

**Authors:** Ahmed Rezk, Nehad Bakry, Samar Elfiky, Maha Metawaa, Ahmed Ibrahim

**Affiliations:** ^1^ Department of Pediatrics, Faculty of Medicine, Ain Shams University, Cairo, Egypt; ^2^ Department of Pediatrics, Faculty of Medicine, Suez Canal University, Ismailia, Egypt; ^3^ Department of Clinical Pathology, Faculty of Medicine, Ain Shams University, Cairo, Egypt

**Keywords:** pediatric pneumonia, salivary biomarkers, interleukin-6, interleukin-10, procalcitonin

## Abstract

**Objectives:**

Effective biomarkers are essential for improving the diagnosis and risk stratification of pediatric pneumonia. This study aimed to evaluate the diagnostic and prognostic utility of salivary and serum interleukin (IL)-6, interleukin (IL)-10, and procalcitonin (PCT) in children diagnosed with pneumonia.

**Methods:**

A prospective case-control study was conducted involving 50 children under five years of age with community-acquired pneumonia (CAP) and 50 age- and sex-matched healthy controls. At admission, serum and saliva samples were collected, and levels of PCT, IL-6, and IL-10 were measured using ELISA. Receiver operating characteristic (ROC) curve analysis was used to evaluate the diagnostic performance of each biomarker in distinguishing children with pneumonia from healthy controls. Multivariate logistic regression was then applied to identify independent predictors of disease severity.

**Results:**

All three biomarkers demonstrated exceptional diagnostic accuracy in distinguishing pneumonia from healthy controls. Salivary PCT (>68.5 pg/ml, AUC = 1.000) and serum IL-10 (>73.18 pg/ml, AUC = 1.000) achieved perfect diagnostic performance with 100% sensitivity and 100% specificity. Serum IL-6 (>18.06 ng/L, AUC = 0.994) and serum PCT (>86.66 pg/ml, AUC = 0.962) also demonstrated excellent accuracy with 96% sensitivity and 100% specificity. The neutrophil-to-lymphocyte ratio (>0.8, AUC = 1.000) similarly achieved 100% sensitivity and specificity. Severe pneumonia was associated with higher IL-10 and PCT levels (both serum and saliva), younger age, elevated heart rate, and higher CRP. IL-6 did not correlate with severity. In multivariate analysis, age <6 months (OR: 3.85), neutrophil-to-lymphocyte ratio (OR: 3.40), serum IL-10 (OR: 5.75), and salivary PCT (OR: 4.25) independently predicted severe pneumonia.

**Conclusions:**

Salivary and serum IL-6, IL-10, and PCT show promising diagnostic potential for pediatric pneumonia when compared to healthy controls. IL-10 and PCT also demonstrate prognostic value for severity stratification, with salivary measurements closely mirroring serum results. While these findings suggest potential for saliva-based diagnostics as non-invasive tools for early detection and severity assessment in pediatric pneumonia, validation in clinical settings with symptomatic controls is needed to establish their practical diagnostic utility in differentiating pneumonia from other febrile illnesses.

## Introduction

Community-acquired pneumonia (CAP) remains a leading cause of mortality among children under five years old worldwide and represents one of the most common pediatric infectious diseases in both developed and developing nations, contributing significantly to antibiotic consumption and hospitalization rates (1).

Accurate assessment of disease severity and timely identification of the causative pathogen are critical for optimizing clinical management in pediatric pneumonia. However, despite advancements in diagnostic technologies, validated prognostic tools remain scarce. Consequently, clinicians often rely on a combination of clinical evaluation, microbiological analysis of sputum, and imaging modalities such as chest radiography and ultrasonography to determine the severity and extent of the disease (2).

Biomarkers reflecting the host's immune response to infection provide objective measures for diagnosing pneumonia and assessing its severity, thereby potentially improving clinical decision-making and prognostication. Among these, interleukin (IL)-6 and interleukin (IL)-10 are key cytokines involved in immune regulation and inflammation. These pleiotropic inflammatory mediators are produced by various cell types following infection (3, 4). Furthermore, IL6 and IL10, are recognized as an early and sensitive indicator of bacterial infections, with substantial evidence supporting its diagnostic utility in severe CAP (5). During the initial phases of pneumonia, alveolar macrophages release inflammatory cytokines such as tumor necrosis factor-α (TNF-α), IL-10, and IL-6. Systemic and bronchoalveolar levels of these cytokines increase as the disease progresses. Therefore, Elevated levels of IL-6 and IL-10 have been linked to a higher risk of mortality in children and severe lung damage (6).

C-reactive protein (CRP), a widely utilized marker of systemic inflammation, has been extensively investigated for its role in the diagnosis and monitoring of pediatric pneumonia (7, 8). Additionally, mean platelet volume (MPV) is recognized as an indicator of platelet activation and potentially serves as an early diagnostic marker for sepsis (9). Other markers, such as the CRP/albumin ratio, neutrophil-to-lymphocyte ratio (NLR), and platelet-to-lymphocyte ratio (PLR), have been assessed in pneumonia research, although data regarding their clinical utility in predicting disease progression remain limited (10).

Procalcitonin (PCT) has re-emerged as a significant biomarker, demonstrating high diagnostic accuracy for the early detection of bacterial infections, including sepsis. Compared to conventional inflammatory markers, PCT offers superior sensitivity and specificity, positioning it as a valuable tool for both diagnosis and monitoring the effectiveness of antimicrobial therapy, particularly in critically ill patients (11).

Several studies highlight the increasing importance of biomarkers in pediatric pneumonia for guiding personalized treatment approaches and predicting disease severity. These biomarkers facilitate the identification of high-risk patients, allowing clinicians to reduce unnecessary interventions, optimize resource allocation, and tailor management plans. In critically ill children, this biomarker-guided approach enhances therapeutic outcomes and diagnostic accuracy (12).

Recent studies indicate that saliva is a promising biofluid for non-invasive infection screening. This method is particularly suitable for pediatric populations due to its patient-friendly nature and ease of collection. Additionally, by lowering the risks and discomfort of venipuncture, the use of salivary diagnostics can increase patient compliance, especially in young children. Salivary biomarkers are non-invasive and have been demonstrated to have a strong correlation with systemic inflammatory responses, offering useful data for the diagnosis and surveillance of a number of infections (8).

This study aimed to evaluate the diagnostic and prognostic significance of PCT, IL-6, and IL-10 in the serum and saliva of pediatric patients with CAP. Moreover, the study aimed to determine the reliability of salivary testing as a non-invasive alternative for early diagnosis and severity prediction in pediatric pneumonia by comparing salivary and serum biomarker levels.

## Materials and methods

### Study design and population

**Figure 1:**
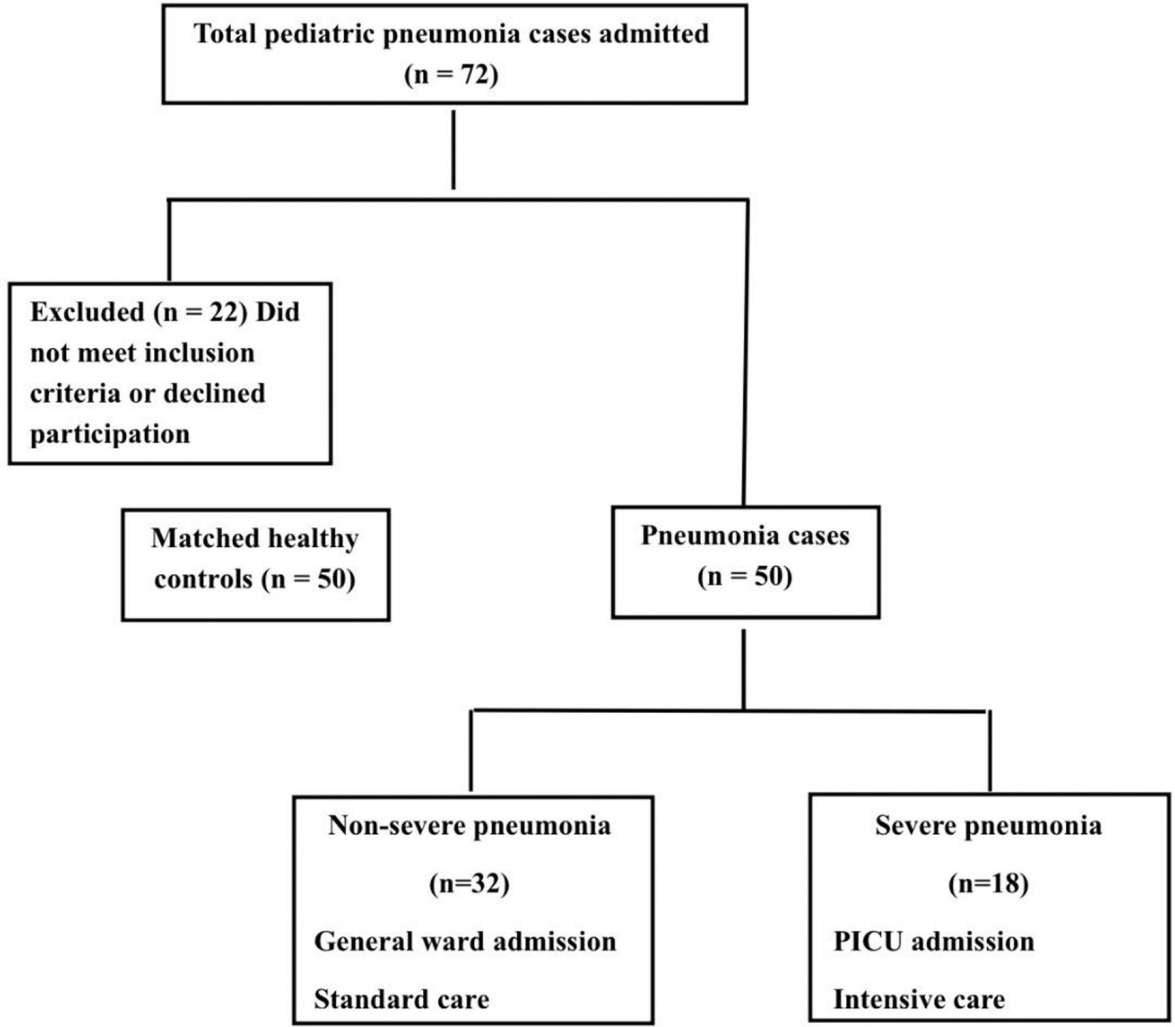
Flow diagram of study participants.

This prospective case-control study was conducted at Ain Shams University Children's Hospital between April 2023 and September 2023. The study enrolled a total of 100 children, comprising 50 children with pneumonia and 50 healthy controls. The study population comprised three groups of children under the age of five years (Figure 1).

Severe pneumonia group: Children admitted to the Pediatric Intensive Care Unit (PICU) with CAP meeting the Infectious Diseases Society of America (IDSA) criteria for severe illness. Admission criteria included respiratory failure necessitating invasive mechanical ventilation via endotracheal intubation or non-invasive positive pressure ventilation (e.g., CPAP or BiPAP), persistent hypoxemia despite high inspired oxygen concentrations (FiO_2_ ≥ 0.50 to maintain SpO_2_ > 92%), hemodynamic instability requiring pharmacologic support, frequent apnea, or altered mental status attributable to hypercarbia or hypoxemia (13).

Non-severe pneumonia group: Children hospitalized for CAP who did not require PICU admission. These patients received standard medical care in general pediatric wards, based on clinical indications such as moderate respiratory distress or need for supportive therapy without meeting criteria for critical care.

Healthy control group: Age- and sex-matched children without signs of acute illness or underlying chronic inflammatory conditions, recruited from outpatient clinics.

The diagnosis of CAP was based on a combination of clinical history, physical examination, and radiographic findings consistent with pneumonia. Common presenting features included fever, cough, tachypnea, and increased work of breathing. On physical examination, retractions, nasal flaring, grunting, and abnormal auscultatory findings (such as crackles or decreased breath sounds) were typically observed (14).

Children with any of the following conditions were excluded: aspiration pneumonia, congenital pulmonary malformations, bronchopulmonary dysplasia, primary or secondary immunodeficiency, malignancy, hematologic disorders (e.g., sickle cell anemia), congenital heart disease with hemodynamic significance, neuromuscular disease affecting respiration, dependence on home ventilation or tracheostomy, chronic corticosteroid or immunosuppressive therapy, or recent major trauma or surgery.

### Sample size calculation

Sample size calculation was performed *a priori* using G*Power 3.1.9.7 (Heinrich Heine University, Düsseldorf, Germany). Based on previous studies of procalcitonin and cytokine levels in pediatric pneumonia (6, 12, 15), we assumed a medium effect size (Cohen's *d* = 0.5) for biomarker differences between cases and controls. With *α* = 0.05 (two-tailed) and power = 0.80, the minimum required sample was 43 participants per group. We enrolled 50 children per group (*n* = 100 total) to account for potential exclusions or missing data.

### Sample collection and laboratory analysis

Serum and saliva samples were collected simultaneously at the time of hospital admission, prior to initiating antibiotic therapy or other clinical interventions, ensuring accurate baseline measurements of biomarkers.

#### Blood sample collection

Two ml of venous blood was collected and separated in plain tubes. Serum was allowed to clot for 10–20 min at room temperature and centrifuged at 2,000–3,000 RPM for 20 min. The supernatant was collected without sediment. Complete blood count (CBC) with differential, MPV, CRP, CRP/MPV ratio, NLR, PLR, and venous blood gas (VBG) are all routinely done.

#### Salivary sample collection

Unstimulated saliva samples were collected 1 h before feeding to avoid contamination. Children were instructed not to speak, move their tongues, or swallow, and their heads were tilted forward to allow saliva to pool in the mouth for approximately 1 min. Using a 1-ml syringe connected to low-wall suction (<20 mm Hg), 0.5 ml of saliva was gently aspirated from beneath the tongue and gingival crevices over 10–15 s. The sample remained in the syringe without entering the tubing or suction trap. After collection, saliva was transferred to polypropylene vials, centrifuged at 4,000 rpm for 10 min, and the supernatant was stored at −20 °C.

#### Biomarker assessment

The serum and salivary PCT, IL-6, and IL10 levels were assessed by an enzyme-linked immunosorbent assay (ELISA) commercial kit (E0090Hu BT lab, China), (E0102Hu BT lab, China) and (E0977 Hu BT lab, China).

### Statistical analysis

The statistical analysis was conducted using the Statistical Package for Social Sciences (SPSS) version 15.0 (SPSS Inc., Chicago, IL). Descriptive statistics were employed to summarize the data. Categorical variables were presented as frequencies and percentages, whereas continuous variables were reported as mean ± standard deviation (SD) or median with interquartile range (IQR), contingent upon the data distribution. Comparisons of continuous variables between groups were conducted using Mann–Whitney *U* tests and independent *t*-tests. Chi-square tests were employed to analyze categorical data. Receiver operating characteristic (ROC) curves were generated to assess the diagnostic significance of the biomarkers. The predictive accuracy of each biomarker was evaluated through the calculation of its area under the curve (AUC), sensitivity, specificity, positive predictive value (PPV), and negative predictive value (NPV). Youden's index was employed to ascertain the optimal cutoff points for serum and salivary concentrations of IL-6, IL-10, PCT, and CRP. Univariate and multivariate logistic regression analyses, employing the Backward Wald method, were performed to identify factors significantly correlated with pneumonia severity. Confidence intervals (CI) at the 95% level for odds ratios (OR) were presented.

## Results

### Diagnostic differentiation: pneumonia vs. healthy Controls

A total of 100 children were enrolled in the study, comprising 50 patients with CAP and 50 healthy controls. There were no significant differences in age (median 14 months in the patient group vs. 16 months in the control group, *p* = 0.899) or sex distribution (males: 55% vs. 58%; females: 46% vs. 42%, in the patient and control groups respectively; *p* = 0.934), indicating effective matching of baseline demographic characteristics (Table 1).

**Table 1:** Demographic, hematological, and inflammatory marker profiles in pediatric pneumonia patients vs. healthy controls.

Parameter		Control group	Patients group	*P*-value	Significance
		No. = 50	No. = 50		
Age (months)	Median (IQR)	16 (6–28)	14 (5–24)	0.899	NS
	Range	3–52	4–54		
Sex	Female	21 (42.0%)	23 (46.0%)	0.934	NS
	Male	29 (58.0%)	27 (55.0%)		
Neutrophils (×10⁹/L)	Median (IQR)	2.1 (1.5–3)	6 (3.5–8.1)	<0.001	HS
	Range	1.5–3.5	1.7–21.3		
Lymphocytes (×10⁹/L)	Mean ± SD	4.71 ± 1.59	4.87 ± 2.08	0.761	NS
	Range	2.5–7	1.9–9.7		
Hemoglobin (g/dl)	Mean ± SD	11.32 ± 0.99	10.02 ± 1.38	<0.001	HS
	Range	9.8–13	7.3–13.9		
Platelet count	Mean ± SD	335.32 ± 99.91	433.80 ± 174.05	0.018	S
	Range	190–450	140–791		
CRP (mg/L)	Median (IQR)	2 (1.7–2.5)	28.3 (4.3–51.9)	<0.001	HS
	Range	1.5–3	0.1–201		
MPV (fL)	Mean ± SD	8.08 ± 2.19	9.31 ± 3.57	0.146	NS
	Range	5–12	1–18		
MPV/CRP	Median (IQR)	3.9 (2.7–6.1)	0.25 (0.12–2.6)	<0.001	HS
	Range	2–6.6	0.03–140		
Neutrophil-lymphocyte ratio	Median (IQR)	0.4 (0.2–0.6)	1.3 (1–2)	<0.001	HS
	Range	0.2–0.8	0.6–4.8		
Platelet-lymphocyte ratio	Median (IQR)	89 (46–220)	97 (58–120)	0.946	NS
	Range	42–266	7–217		
Serum IL-10 pg/ml	Median (IQR)	66.03 (57.45–67.49)	379.1 (265.7–602.8)	<0.001	HS
	Range	54.42–73.18	95.93–1,600		
Serum IL-6 ng/L	Median (IQR)	13.14 (9.69–17.19)	47.37 (32.63–61.63)	<0.001	HS
	Range	9.54–18.06	17.86–108.5		
Serum procalcitonin pg/ml	Median (IQR)	61.73 (59.06–73.93)	568.6 (224–871.3)	<0.001	HS
	Range	43.98–86.66	43.98–1,082		
Salivary IL-10 pg/ml	Median (IQR)	64.38 (63.51–74.12)	212.8 (84.36–384)	<0.001	HS
	Range	52.93–77.6	63.34–1,600		
Salivary IL-6 ng/L	Median (IQR)	15.42 (10.99–18.17)	28.67 (14.34–77.84)	<0.001	HS
	Range	7.74–18.41	8.3–128.6		
Salivary procalcitonin pg/ml	Median (IQR)	54.82 (47.27–64.51)	325.1 (235.4–765.8)	<0.001	HS
	Range	46.31–68.54	124.2–2,400		

CRP, C-reactive protein; MPV, mean platelet volume; IQR, interquartile range; NS, non-significant; S, significant; HS, highly significant.

Children with pneumonia demonstrated pronounced abnormalities in routine hematological parameters compared to healthy children. Neutrophil counts were markedly elevated in patients (median 6.0 × 10^9^/L, IQR: 3.5–8.1) vs. controls (median 2.1 × 10^9^/L, IQR: 1.5–3.0), a highly significant difference (*p* < 0.001). Conversely, the lymphocyte counts were similar between the two groups (mean 4.87 vs. 4.71 × 10^9^/L, *p* = 0.761). Children with pneumonia had significantly lower hemoglobin levels (mean 10.02 ± 1.38 g/dl) than controls (11.32 ± 0.99 g/dl, *p* < 0.001), and a higher platelet count (mean 433.8 ± 174 × 10^9^/L vs. 335.3 ± 99.9 × 10^9^/L, *p* = 0.018), consistent with an acute inflammatory response in infection.

Inflammatory markers were notably elevated in children with pneumonia. CRP, a key systemic inflammation marker, was significantly higher in patients (median: 28.3 mg/L, IQR: 4.3–51.9) compared to controls (median: 2.0 mg/L, IQR: 1.7–2.5; *p* < 0.001). The NLR was also substantially greater in children with pneumonia (median: 1.3, IQR: 1.0–2.0) vs. healthy children (median: 0.4, IQR: 0.2–0.6; *p* < 0.001), reflecting an infection-driven shift in leukocyte subsets. In contrast, the PLR did not differ between groups (*p* = 0.946), suggesting it may be less useful in this context (Table 1).

Inflammatory cytokine levels showed significant increases in children with pneumonia relative to controls, in both serum and saliva (Table 1). Median serum IL-10 was 379.1 pg/ml (IQR: 265.7–602.8) in children with pneumonia, dramatically higher than 66.03 pg/ml (IQR: 57.45–67.49) in healthy controls (*p* < 0.001). Similarly, serum IL-6 was elevated in patients (median: 47.37 ng/L, IQR: 32.63–61.63) compared to controls (median: 13.14 ng/L, IQR: 9.69–17.19; *p* < 0.001). Serum PCT showed the most pronounced difference, with a median of 568.6 pg/ml (IQR: 224.0–871.3) in children with pneumonia vs. 61.73 pg/ml (IQR: 59.06–73.93) in controls (*p* < 0.001). Salivary biomarkers reflected these trends: salivary IL-10, IL-6, and PCT were all significantly higher in children with pneumonia than in healthy children (all *p* < 0.001). These findings indicate that both serum and saliva levels of IL-6, IL-10, and PCT robustly distinguish children with pneumonia from healthy controls.

### ROC curve analysis for pneumonia diagnosis

**Figure 2:**
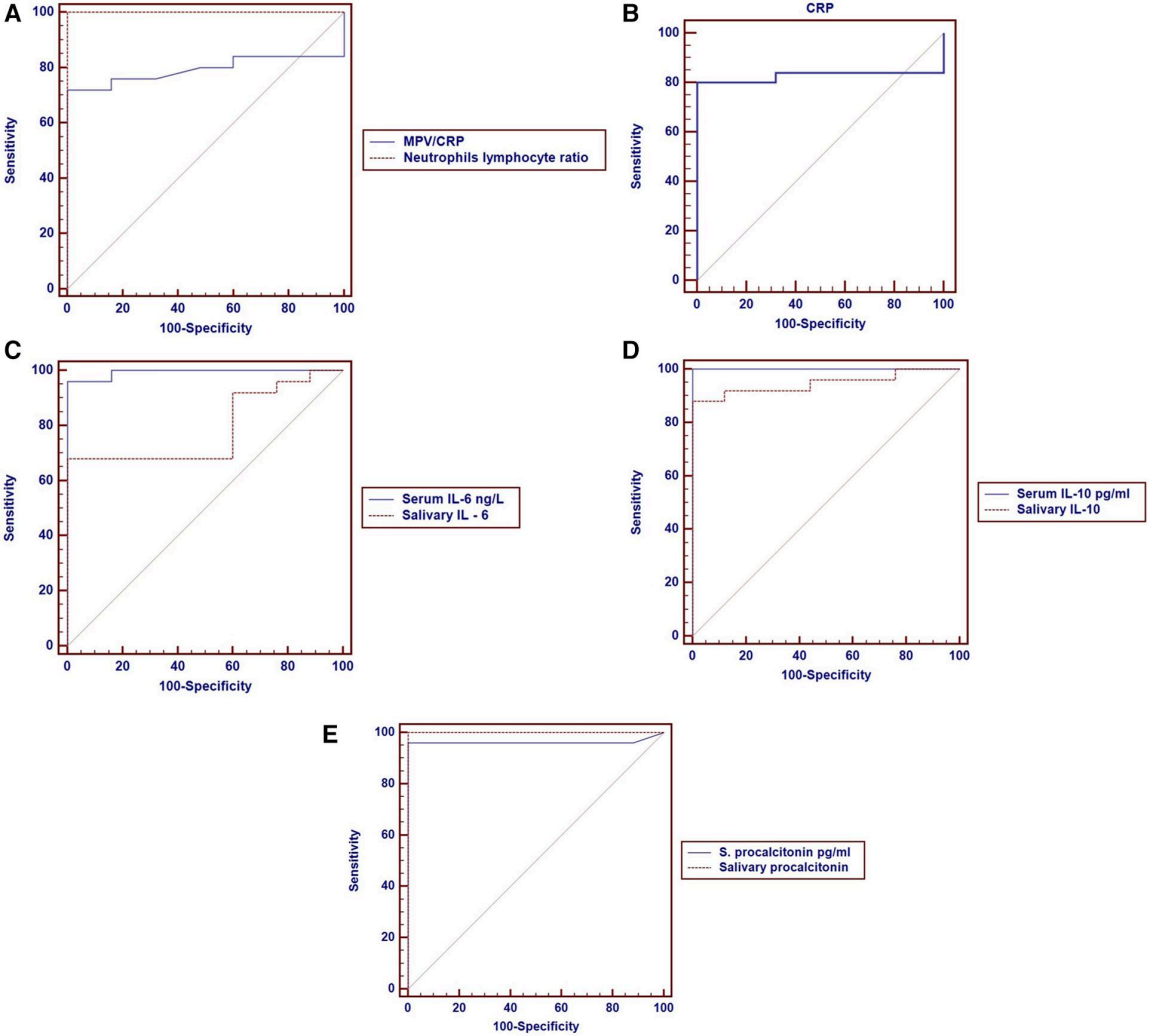
Receiver operating characteristic (ROC) curve analyses of various biomarkers as predictors of pneumonia. **(A)** Presents MPV/CRP and neutrophil-to-lymphocyte ratio (NLR). MPV/CRP showed an AUC of 0.794 with a cut-off ≤0.9 (sensitivity 72.0%, specificity 100.0%), while NLR achieved an AUC of 1.000 at a cut-off >0.8 (sensitivity and specificity 100.0%). **(B)** Shows CRP with an AUC of 0.827 at a cut-off >3.0 (sensitivity 80.0%, specificity 100.0%). **(C)** Includes serum IL-6 with an AUC of 0.994 (cut-off >18.06 ng/L, sensitivity 96.0%, specificity 100.0%) and salivary IL-6 with an AUC of 0.790 (cut-off >18.41 ng/L, sensitivity 68.0%, specificity 100.0%). **(D)** Displays serum IL-10 with an AUC of 1.000 (cut-off >73.18 pg/ml, sensitivity and specificity 100.0%) and salivary IL-10 with an AUC of 0.947 (cut-off >77.6 pg/ml, sensitivity 88.0%, specificity 100.0%). **(E)** Presents serum procalcitonin with an AUC of 0.962 (cut-off >86.66 pg/ml, sensitivity 96.0%, specificity 100.0%) and salivary procalcitonin with an AUC of 1.000 (cut-off >68.54 pg/ml, sensitivity and specificity 100.0%).

ROC curve analysis further quantified the diagnostic accuracy of each biomarker for identifying pneumonia (Figures 2A–E). Remarkably, several markers achieved near-perfect discrimination between pneumonia and health. The optimal cut-off for NLR (>0.8) yielded 100% sensitivity and 100% specificity, correctly classifying all patients and controls (Figure 2A). Likewise, a salivary PCT threshold >68.5 pg/ml (Figure 2E) and a serum IL-10 threshold >73.18 pg/ml (Figure 2D) each demonstrated 100% sensitivity and 100% specificity for pneumonia diagnosis, underscoring their exceptional performance. Serum PCT >86.66 pg/ml (Figure 2E) and serum IL-6 > 18.06 ng/L (Figure 2C) also showed excellent diagnostic power, with 96.0% sensitivity and 100.0% specificity for each, further supporting their clinical relevance in detecting pneumonia. Salivary IL-10 >77.6 pg/ml (Figure 2D) achieved 100% specificity with a slightly lower sensitivity (88.0%), indicating that while nearly all healthy children had salivary IL-10 below this level, a few milder pneumonia cases fell below the cutoff. CRP >3 mg/L (Figure 2B), a traditional marker, exhibited strong specificity (100%) but only moderate sensitivity (80.0%).

### Prognostic stratification: severe vs. non-severe pneumonia

Among the 50 children with pneumonia, 18 (36%) had severe pneumonia requiring PICU admission, while 32 (64%) had non-severe pneumonia managed on general wards (Figure 1). Clinical comparisons between these subgroups revealed key differences linked to disease severity (Table 2). Children with severe pneumonia were significantly younger than those with non-severe illness (median age 8 months, IQR: 3–11 vs. 17 months, IQR: 3–24; *p* = 0.036), suggesting that infants are at higher risk for developing life-threatening pneumonia. Severe cases also had a higher heart rate on admission (mean: 159.6 ± 17.95 bpm) compared to non-severe cases (142.3 ± 16.36 bpm; *p* = 0.021), consistent with greater systemic stress in critically ill children. In contrast, respiratory rate did not differ significantly between severe and non-severe groups (mean: 50.0 vs. 46.1 breaths/min, *p* = 0.389). As expected, the length of hospital stay was markedly longer for severe pneumonia (median: 12 days, IQR: 10–14) than for milder cases (median: 5 days, IQR: 4–7; *p* < 0.001), reflecting the increased care needs of critically ill patients. There were no significant differences in the microbiological findings between the two groups: the rates of respiratory viral panel positivity (50% vs. 12.5%, *p* = 0.065) and bacterial growth on sputum culture (38.9% vs. 21.9%, *p* = 0.275) were statistically similar, indicating that pathogen profile alone did not explain the severity differences in our cohort.

**Table 2:** Demographic and clinical parameters associated with pneumonia severity in hospitalized pediatric patients.

Parameter		Non-severe pneumonia	Severe pneumonia	*P*-value	Significance
		No. = 32	No. = 18		
Age (months)	Median (IQR)	17 (3–24)	8 (3– 11)	0.036	S
	Range	6–58	3– 50		
Sex	Female	14 (43.7%)	9 (50.0%)	0.742	NS
	male	18 (56.3%)	9 (50.0%)		
Heart Rate (bpm)	Mean ± SD	142.33 ± 16.36	159.60 ± 17.95	0.021	S
	Range	110–170	137–180		
Respiratory Rate (bpm)	Mean ± SD	46.13 ± 9.99	50.00 ± 11.93	0.389	NS
	Range	31–66	32–66		
Admission (days)	Median (IQR)	5 (4–7)	12 (10–14)	<0.001	HS
	Range	3–12	8–18		
Respiratory viral panel (Bio-fire)	No growth (No, %)	28 (87.5)	9 (50)	0.065	NS
	Positive (No, %)	4 (12.5)	9 (50)		
Sputum culture	No growth (No, %)	25 (78.1)	11 (61.1)	0.275	NS
	Positive (No, %)	7 (21.9)	7 (38.9)		
Fate	Discharged (No, %)	32 (100)	16 (88.9)	0.342	NS
	Died (No, %)	0 (0)	2 (11.1)		

Bpm, beat per minute; interquartile range; NS, non-significant; S, significant; HS, highly significant.

### Laboratory markers and severity prediction

Several laboratory markers were associated with pneumonia severity (Table 3). CRP levels were higher in severe cases (median: 46.8 mg/L, IQR: 12.5–60.8) compared to non-severe cases (27.0 mg/L, IQR: 2.3–51.9; *p* = 0.034), consistent with a more intense inflammatory response in severe infection. Additionally, the median NLR was higher in the severe pneumonia group (1.9, IQR: 1.0–2.9) than in the non-severe group (1, IQR: 0.8–1.6; *p* = 0.032). Other indices such as the platelet-to-lymphocyte ratio (PLR) and mean platelet volume (MPV) showed no significant differences between severe and non-severe pneumonia (*p* > 0.05 for both), suggesting they were not strong determinants of severity in our sample. Both serum IL-10 and serum PCT were significantly elevated in children with severe pneumonia. Median serum IL-10 in severe cases was 467.45 pg/ml (IQR: 293.4–1,073) vs. 280.0 pg/ml (IQR: 235.2–509.4) in non-severe cases (*p* = 0.0323). Similarly, median serum PCT was 672.5 pg/ml (IQR: 238.1–871.3) in severe pneumonia, compared to 269.15 pg/ml (IQR: 172.7–998.7) in non-severe pneumonia (*p* = 0.045). This same pattern was reflected in saliva: the severe group had higher salivary IL-10 (median: 336.1 vs. 184.5 pg/ml, *p* = 0.0437) and higher salivary PCT (median: 452.4 vs. 245.95 pg/ml, *p* = 0.021) than the non-severe group. In contrast, IL-6 levels did not significantly differ between severe and non-severe pneumonia in either serum or saliva (*p* > 0.47), indicating that IL-6 elevation occurs in pneumonia but was not a distinguishing factor for progression to critical illness in this cohort. Together, these subgroup comparisons suggest that while all three markers (IL-6, IL-10, PCT) rise in the presence of pneumonia, only IL-10 and PCT levels (in both serum and saliva) correlated with disease severity, whereas IL-6 did not.

**Table 3:** Laboratory parameters associated with pneumonia severity in hospitalized pediatric patients.

Parameter		Non-severe pneumonia	Severe pneumonia	*P*-value	Significance
		No. = 32	No. = 18		
CRP (mg/L)	Median (IQR)	27 (2.3–51.9)	46.8 (12.5–60.8)	0.034	S
	Range	0.5–127	0.1–201		
MPV (fL)	Mean ± SD	9.23 ± 2.92	9.43 ± 4.55	0.896	NS
	Range	3.1–15.2	1–18		
MPV/CRP	Median (IQR)	0.3 (0.13–4.3)	0.19 (0.04–0.8)	0.211	NS
	Range	0.06–24	0.03–140		
Neutrophil-lymphocyte ratio	Median (IQR)	1 (0.8–1.6)	1.9 (1–2.9)	0.032	S
	Range	0.6–4.8	1–4.5		
Platelet-lymphocyte ratio	Median (IQR)	99.5 (60–120)	75.5 (44–120)	0.488	NS
	Range	42–217	7–163		
Serum IL-10 pg/ml	Median (IQR)	280 (235.2–509.4)	467.45 (293.4–1,073)	0.0323	S
	Range	95.93–1,600	176.2–1,600		
Serum IL-6 ng/L	Median (IQR)	50.9 (32.74–62.96)	40.39 (18.62–61.63)	0.471	NS
	Range	17.86–108.5	18.17–91.15		
Serum procalcitonin pg/ml	Median (IQR)	269.15 (172.7–998.7)	672.5 (238.1–871.3)	0.045	S
	Range	114.9–1,044	43.98–1,082		
Salivary IL-10 pg/ml	Median (IQR)	184.5 (96.14–277.2)	336.1 (84.16–569.8)	0.0437	S
	Range	63.34–680.3	69.08–1,600		
Salivary IL-6 ng/L	Median (IQR)	28.31 (14.02–54.98)	53.26 (14.34–82.93)	0.471	NS
	Range	8.3–108.8	10.23–128.6		
Salivary procalcitonin pg/ml	Median (IQR)	245.95 (154.2–765.8)	452.4 (298.4–1,047)	0.021	S
	Range	124.2–1,063	200.2–2,400		

CRP, C-reactive protein; MPV, mean platelet volume; IQR, interquartile range; NS, non-significant; S, significant; HS, highly significant.

### Multivariate analysis of severity predictors

To identify independent predictors of severe pneumonia, a multivariate logistic regression analysis was performed (Table 4). The model highlighted four factors that were significantly associated with higher odds of PICU admission for pneumonia. Age below 6 months was a strong independent risk factor for severe disease (adjusted OR = 3.85, 95% CI: 1.90–7.80, *p* = 0.002), underscoring the vulnerability of young infants. An elevated neutrophil-to-lymphocyte ratio also emerged as an independent predictor (OR = 3.40 per unit increase in NLR, 95% CI: 1.75–6.85, *p* = 0.003), reflecting the role of an exaggerated immune response in severe outcomes. Among the biomarkers, high serum IL-10 was the most powerful cytokine predictor of severity (OR = 5.75, 95% CI: 2.80–11.70, *p* < 0.001), indicating that children with markedly elevated IL-10 had nearly six-fold higher odds of developing severe pneumonia. Notably, salivary PCT was identified as an independent non-invasive marker of severity (OR = 4.25, 95% CI: 2.05–8.50, *p* < 0.001): even after adjusting for other factors, elevated PCT in saliva was associated with over four-fold increased odds of a severe disease course. These four predictors remained significant in the final multivariate model, whereas other variables (such as IL-6 levels, CRP, heart rate, etc.) did not retain independent significance.

**Table 4:** Multivariate logistic regression analysis identifying independent predictors of severe pneumonia requiring PICU admission.

Variable	Adjusted OR	(95% CI)		*P*-value	Significance
		Lower	Upper		
Age below 6 months	3.85	1.90	7.80	0.002	S
Neutrophil-lymphocyte Ratio	3.40	1.75	6.85	0.003	S
Serum IL-10 (pg/ml)	5.75	2.80	11.70	<0.001	HS
Salivary Procalcitonin (pg/ml)	4.25	2.05	8.50	<0.001	HS

OR, odds ratio; CI, confidence interval; NS, non-significant; S, significant; HS, highly significant.

## Discussion

### Diagnostic performance of inflammatory biomarkers

The inflammatory response in children with pneumonia was characterized by significant elevations in multiple biomarkers compared to healthy controls, demonstrating robust systemic immune activation associated with this condition. CRP, a well-established acute-phase reactant, showed strong diagnostic utility with significantly higher levels in pneumonia cases (median: 28.3 mg/L, IQR: 4.3–51.9) vs. controls (median: 2.0 mg/L, IQR: 1.7–2.5; *p* < 0.001), confirming its established role as a sensitive marker for pneumonia diagnosis (16). Consistently elevated or rising CRP levels during treatment may indicate antibiotic failure or infectious complications, requiring clinical reassessment and treatment modifications (17).

The NLR demonstrated exceptional discriminatory capacity, with substantially higher values in children with pneumonia (median: 1.3, IQR: 1.0–2.0) vs. healthy children (median: 0.4, IQR: 0.2–0.6; *p* < 0.001), reflecting the characteristic immune response shift during bacterial infections (18). Notably, an NLR threshold >0.8 achieved optimal diagnostic performance with 100% sensitivity and specificity, underscoring its precision in identifying pneumonia cases.

PCT emerged as a particularly powerful diagnostic biomarker, with serum levels markedly elevated in children with pneumonia (median: 568.6 pg/ml, IQR: 224.0–871.3) compared to controls (median: 61.73 pg/ml, IQR: 59.06–73.93; *p* < 0.001). PCT is predominantly synthesized by parenchymal cells in response to bacterial endotoxins and pro-inflammatory cytokines such as IL-6 and TNF-α (19). Consistent with our findings, Ozbay et al. demonstrated that elevated PCT levels effectively distinguish bacterial from nonbacterial pulmonary infections, offering superior diagnostic accuracy compared to conventional markers such as ESR and CRP (20). In our ROC analysis, a PCT threshold of >86.66 pg/ml achieved 96% sensitivity and 100% specificity, underscoring its robust diagnostic performance. Although a meta-analysis by Tsou et al. reported only moderate pooled sensitivity (0.64) and specificity (0.72) for PCT in pediatric pneumonia (21), our higher accuracy may reflect differences in study design, patient selection, or the application of optimized thresholds tailored to a well-defined population.

The cytokine profile analysis provided additional diagnostic insights. Serum IL-6 levels were significantly elevated in children with pneumonia (median: 47.37 ng/L, IQR: 32.63–61.63) compared to controls (median: 13.14 ng/L, IQR: 9.69–17.19; *p* < 0.001), with a threshold of >18.06 ng/L achieving 96% sensitivity and 100% specificity. As a central mediator of the acute-phase response, IL-6 promotes CRP synthesis in hepatocytes, enhances neutrophil activation, and regulates the early inflammatory cascade by mobilizing immune cells to the site of infection (22). Elevated IL-6 in children with pneumonia reinforce its value as a sensitive biomarker of systemic inflammation and highlight its pathophysiological relevance in pneumonia (23).

IL-10 demonstrated exceptional diagnostic performance with significantly elevated serum levels in pneumonia cases and optimal discrimination at a threshold >73.18 pg/ml (100% sensitivity and specificity). This high accuracy likely reflects IL-10's role in early immune response regulation, where it modulates lung inflammation, neutrophil recruitment, and cytokine signaling pathways (24, 25). While previous studies reported variable diagnostic performance for IL-10 alone, evidence increasingly supports its utility, particularly in combination with other mediators. These findings are supported by Zhao et al., who reported improved diagnostic performance when IL-10 was combined with IL-6 in Mycoplasma pneumoniae pneumonia (26).

### Salivary biomarkers: a non-invasive alternative

Children with pneumonia exhibited significantly higher salivary concentrations of PCT, IL-6, and IL-10 compared to healthy controls, with strong correlations observed between salivary and serum levels. Salivary PCT demonstrated optimal diagnostic performance at a threshold >68.5 pg/ml (100% sensitivity and specificity), while salivary IL-10 showed excellent specificity (100%) with good sensitivity (88%) at >77.6 pg/ml. The comparable diagnostic accuracy between salivary and serum biomarkers is consistent with evidence that salivary cytokines accurately mirror systemic inflammatory responses (27). In pediatric practice, saliva offers significant advantages as a non-invasive, painless, and easily repeatable sampling method that reduces patient discomfort and procedural complications while improving compliance and clinical efficiency (28). These attributes support the practical and cost-effective application of salivary biomarkers for diagnostic and surveillance purposes, positioning them as clinically viable alternatives to serum testing for pediatric pneumonia diagnosis and monitoring.

### Prognostic value and severity stratification

Accurate severity stratification is crucial for optimal resource allocation and patient management. In our study, infants with severe pneumonia were significantly younger than those with non-severe disease (median: 8 vs. 17 months; *p* = 0.036). Furthermore, on multivariable logistic regression, age <6 months independently predicted severe pneumonia requiring PICU admission (adjusted OR: 3.85; 95% CI: 1.90–7.80; *p* = 0.002). This age-related vulnerability is well documented and likely reflects anatomic and immunologic immaturity, attenuated T-cell responses, reduced cytokine production, and impaired neutrophil chemotaxis (29) together with limited pulmonary reserves, reduced airway caliber, and impaired mucociliary clearance, which facilitate rapid deterioration (13, 30).

The prognostic utility of inflammatory biomarkers was particularly evident in the severity analysis. Children with severe pneumonia exhibited significantly higher CRP levels (median: 46.8 mg/L, IQR: 12.5–60.8) compared to those with non-severe disease (median: 27.0 mg/L, IQR: 2.3–51.9; *p* = 0.034). CRP is a recognized acute-phase reactant, with previous studies indicating a significant correlation between increased complication risk and severe bacterial pneumonia when CRP levels exceed 40 mg/L (31). Similarly, serum PCT levels were markedly elevated in severe cases (672.5 vs. 269.15 pg/ml, *p* = 0.045), supporting its established role as a prognostic marker in pediatric CAP. Yadav et al. conducted a cohort study that agreed our findings, evaluating the predictive value of serum CRP and PCT levels in children with CAP. The study indicated that in hospitalized pediatric patients with CAP, both biomarkers are effective in evaluating disease severity and outcomes. The study found a significant association between elevated serum PCT levels and increased disease severity, thereby endorsing the use of PCT as a prognostic marker in pediatric CAP (32). NLR emerged as a significant predictor of pneumonia severity in our research. Children diagnosed with severe pneumonia exhibited a median NLR of 1.9 (IQR: 1.0–2.9), which was significantly higher than the median NLR of 1.0 (IQR: 0.8–1.6) observed in those with non-severe disease (*p* = 0.032). Multivariate analysis indicated that NLR was an independent risk factor (OR: 3.40, 95% CI: 1.75–6.85, *p* = 0.003). These findings align with previous research emphasizing the significance of the NLR as a marker of systemic inflammation and adverse outcomes in pediatric pneumonia. In accordance with these results, Kuikel et al. conducted a comprehensive study demonstrating that elevated NLR values are consistently associated with increased disease severity and a higher risk of mortality in cases of CAP (33).

Elevated serum IL-10 levels were significantly correlated with increased disease severity (OR: 5.75, 95% CI: 2.80–11.70, *p* < 0.001). Cytokine profiling in this study indicated that patients with severe pneumonia exhibited significantly higher serum IL-10 levels compared to those with non-severe disease (median: 467.45 pg/ml vs. 280 pg/ml, *p* = 0.032). This finding aligns with the established role of IL-10 as a key anti-inflammatory cytokine that regulates immune responses to avert excessive tissue damage. An exaggerated IL-10 response during severe bacterial infections can compromise the protective anti-inflammatory pathways necessary for effective pathogen clearance, thereby promoting immune dysregulation and worsening clinical outcomes (34). Similar findings showed that higher IL-10 levels at admission were associated with more severe bacterial pneumonia with prolonged hospitalization and higher mortality risk (35). Our study found a notable link between high salivary IL-10 levels and severe pneumonia (*p* = 0.0437). Similarly, a study on patients with COVID-related pneumonia demonstrated that saliva could serve as an alternative specimen for assessing IL-10, which showed particular promise as a non-invasive indicator of disease severity (36).

Most significantly, salivary PCT emerged as an independent predictor of severe pneumonia, demonstrating comparable prognostic performance to traditional serum biomarkers. This finding represents a paradigm shift toward non-invasive severity assessment, eliminating procedural challenges associated with repeated venipuncture in critically ill children. The prognostic value of salivary PCT aligns with emerging evidence that infection-related biomarkers in saliva accurately reflect systemic disease processes, providing clinicians with practical tools for real-time risk stratification (37).

### Clinical implications and future directions

The simultaneous assessment of multiple biomarkers across serum and salivary matrices provides an integrated evaluation of systemic inflammatory responses that advances beyond single-biomarker approaches. The validation of salivary biomarkers for diagnosing and risk-stratifying pneumonia could transform pediatric care, particularly in resource-limited settings where non-invasive, point-of-care testing is essential. Future research should focus on validating these biomarkers in larger, multi-center cohorts with diverse patient populations and developing standardized protocols for saliva collection and processing. The development of rapid diagnostic platforms and multiplex assays, combined with machine learning approaches for integrating multiple biomarkers, could further enhance diagnostic accuracy and clinical utility.

### Study limitations

Several limitations should be acknowledged. The single-center design and relatively small sample size, particularly for severe pneumonia cases (*n* = 18), may limit the generalizability of our findings. The use of healthy controls, while appropriate for establishing baseline biomarker performance, does not fully reflect the clinical challenge of differentiating pneumonia from other febrile illnesses in symptomatic children. This design may overestimate diagnostic accuracy compared to real-world clinical scenarios where pneumonia must be distinguished from other respiratory infections. The case-control design precluded assessment of longitudinal biomarker dynamics and treatment response monitoring. Additionally, potential confounding factors such as nutritional status, concurrent medications, or subclinical conditions were not systematically evaluated. Future studies should incorporate symptomatic control groups, larger multicenter cohorts, and longitudinal sampling to better establish clinical utility and validate these promising findings.

## Conclusion

This study demonstrates that salivary and serum biomarkers, particularly PCT, IL-6, and IL- 10, offer significant diagnostic and prognostic value in pediatric pneumonia. The comparable accuracy of salivary biomarkers to their serum counterparts represents a paradigm shift toward non-invasive pediatric diagnostics, with particular relevance for resource-limited settings and scenarios requiring repeated monitoring. While these findings establish proof-of-concept for biomarker-guided pneumonia management, validation in symptomatic control populations is essential to confirm clinical utility in real-world diagnostic scenarios. The integration of multiple biomarkers into standardized diagnostic algorithms could significantly improve early detection, severity assessment, and therapeutic decision-making in pediatric pneumonia care.

## Data Availability

The raw data supporting the conclusions of this article will be made available by the authors, without undue reservation.
